# Multiepitope Subunit Peptide-Based Nanovaccine against Porcine Circovirus Type 2 (PCV2) Elicited High Antibody Titers in Vaccinated Mice

**DOI:** 10.3390/molecules28052248

**Published:** 2023-02-28

**Authors:** Viet Tram Duong, Prashamsa Koirala, Sung-Po R. Chen, Michael J. Monteiro, Mariusz Skwarczynski, Istvan Toth

**Affiliations:** 1 School of Chemistry and Molecular Biosciences, The University of Queensland, St Lucia, QLD 4072, Australia; 2 Australian Institute of Bioengineering and Nanotechnology, The University of Queensland, St Lucia, QLD 4072, Australia; 3 School of Pharmacy, The University of Queensland, Woolloongabba, QLD 4102, Australia

**Keywords:** peptide-based vaccine, adjuvant, polyleucine, liposome, nanoparticles, block copolymers, rods, poly(methyl acrylate), porcine circovirus

## Abstract

Porcine circovirus 2 (PCV2) infection is one of the most serious threats to the swine industry. While the disease can be prevented, to some extent, by commercial PCV2a vaccines, the evolving nature of PCV2 necessitates the development of a novel vaccine that can compete with the mutations of the virus. Thus, we have developed novel multiepitope vaccines based on the PCV2b variant. Three PCV2b capsid protein epitopes, together with a universal T helper epitope, were synthesized and formulated with five delivery systems/adjuvants: complete Freund’s adjuvant, poly(methyl acrylate) (PMA), poly(hydrophobic amino acid), liposomes and rod-shaped polymeric nanoparticles built from polystyrene-poly(*N*-isopropylacrylamide)-poly(*N*-dimethylacrylamide). Mice were subcutaneously immunized with the vaccine candidates three times at three-week intervals. All vaccinated mice produced high antibody titters after three immunizations as analyzed by the enzyme-linked immunosorbent assay (ELISA), while mice vaccinated with PMA-adjuvanted vaccine elicited high antibody titers even after a single immunization. Thus, the multiepitope PCV2 vaccine candidates designed and examined here show strong potential for further development.

## 1. Introduction

Porcine circovirus 2 (PCV2) is a single-stranded DNA virus. Infection with PCV2 leads to porcine multisystemic wasting syndrome (PMWS), that has been considered one of the most serious threats to the swine industry since 1985. PCV2 infection causes a wide range of clinical symptoms, including post-weaning diarrhea, respiratory dyspnea, anemia, icterus and wasting disease, whereby pigs’ growth is hampered. Respiratory distress, tremors, enteric disease, dermatitis, nephropathy and reproductive failure have also been observed. Morbidity rates at affected farms have been estimated to range from 4% to 30% (and occasionally extend to 50–60%), with mortality between 4–20% [1]. The main PCV2 genotypes are labeled PCV2a–h [2]. These eight genotypes share two similar major structural proteins: replicase and capsid. Ongoing genetic mutations have continuously introduced new viral genotypes, including recombinants with changes predominantly on the capsid genetic sequence [3,4]. Analysis of clinical PCV2 cases revealed the most relevant genotypes to be a, b and d [2,5]. There is no direct treatment for PCV2 infection in pigs, and thus vaccination is the only available option to prevent the infection. Moreover, such treatment, with help of monoclonal antibodies, for example, will most likely not be commercially feasible. Consequently, several vaccines were developed and commercialized (Table 1). Current commercial vaccines are derived from the PCV2a genotype including: Ingelvac CircoFLEX^®^ (Boehringer Ingelheim), Circumvent^®^ (Intervet/Merck) and Porcilis^®^ PCV (Schering-Plough/Merck). The capsid protein has been selected as the primary target for vaccine development as the immune responses generated against this protein can neutralize the virus [6,7]. The commercial capsid-based vaccines differ in dosing and efficacy, and these differences are related to the selection of adjuvants used in the formulations (Table 1).

The success of PCV2a vaccines can be attributed to common epitopes shared between the field genotypes and existing PCV2a vaccines. However, as mutations occurring in the capsid sequence accumulated over time, the shared epitopes among PCV2a-based vaccines and field strains declined. Consequently, the distorted immune recognition enabled the virus to escape from the PCV2a vaccine-induced immune response. Thus, the efficacy of the PCV2a vaccines decreased gradually. Many investigations have suggested that, while monovalent PCV2 vaccines provide good homologous protection, bivalent PCV2 vaccines may be the answer to rectify current PCV2a vaccines’ “leaky” nature [8,9]. Thus PCV2a, PCV2b and a universal helper T cell epitope have been combined into one bivalent vaccine to compare its efficacy with a similar monovalent PCV2a counterpart [10]. Animals treated with the bivalent vaccine had a reduced number of PCV2 in their blood and feces compared to the monovalent treated group. Thus, the introduction of PCV2b antigens into the vaccine formulation has been proven to broaden the ability of pigs to respond appropriately to diverse PCV2 field strains.

Due to their success in reducing the prevalence and severity of infection, PCV2a vaccines became the exemplar in the swine industry. However, as mutations in the capsid sequence occur and accumulate over time, the number of shared epitopes between PCV2a-based vaccines and field strains has declined. In fact, alteration in the capsid amino acid sequence changed the epitope conformation, affecting the binding capacity of neutralizing antibodies induced by PCV2a vaccines. The resulting distorted immune recognition enabled the virus to escape from the vaccine-induced immune response [3,9,11,12]. As a result, the efficacy of the PCV2a vaccines decreased gradually.

The inability of current PCV2a vaccines to provide complete cross-protection has prompted researchers to broaden antigenic selection for vaccine design [9]. This study aimed to test the efficacy of a peptide-based subunit vaccine comprised of three PCV2b capsid peptides and one universal helper T cell epitope with novel delivery systems in eliciting antibodies in mice. Peptide-based vaccines contain only the necessary short antigens, thus reducing the chances of adverse reactions [13,14]. These vaccines also provide high vaccine viability and more feasible transport and storage conditions. Peptide-based vaccines can be produced effectively at large scale in a manner similar to, for example, cyclosporine peptide drug manufacturing [15]. However, the antigens commonly incorporated into subunit vaccines are poorly immunogenic [16]. To induce strong, long-lasting immune responses, peptide-based vaccines require an adjuvant (immune stimulator) and multiple immunizations (boosts) [17,18,19].

Many antiviral therapies aim to block the interaction of the virus with receptors and inhibit viral replication by immitating the viral external structures, such as spike proteins [20]. This method has been used successfully in the development of multi-epitope subunit vaccine against the SARS-CoV-2 variants [21]. Following this strategy, we developed our vaccine based on the PCV2 outer envolope, the capsid. Three PCV2b capsid protein-derived peptide sequences (Cap41 [SRTIGYTVKKTTVRTPSWNVDM], Cap67 [SNPLTVPFEYYRIRKVKVE] and Cap121 [KPVLDRTIDYFQPNNKR]) were chosen to enhance the coverage of PCV2b genotypes, as increased breadth of coverage has been shown to consistently improve protective efficacy [10,22]. The selected epitopes (CAP41, CAP67 and CAP121) have been shown to induce the production of IgG in pigs against recombinant CAP OVA PCV2b protein [22]. Incorporation of CD4+ T cell epitope into the vaccine is also crucial to enhance its immunogenicity [23]. Therefore, universal CD4+ T cell epitope P25 (KLIPNASLIENCTKAEL) derived from the fusion protein of the Morbillivirus, canine distemper virus, was included to support uniform humoral responses. The epitope efficacy was proven in previous studies on fertility control, Group A Streptococcus (GAS) and hookworm vaccines [24,25,26,27]. Polylysine (KK) solubilizing moiety was added to each peptide sequence to improve the hydrophilicity of the overall structure.

**Figure 1:**
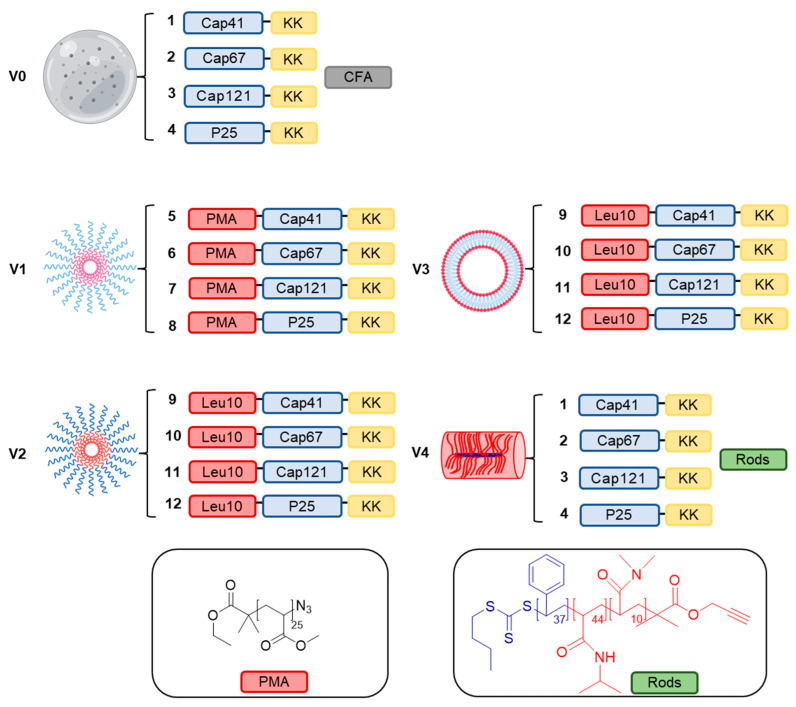
The composition of PCV2b subunit peptide-based vaccine candidates and structure of polymers PMA, Leu10, and rods. Positive control **V0** was formed by emulsification of **1–4** with CFA. Vaccine candidate **V1** was formed through conjugation of **1–4** with PMA to produce **5–8,** respectively. Vaccine candidate **V2** was formed by conjugation of **1–4** with Leu10 (10 repeating units of leucine amino acid) to form **9–12,** respectively. Vaccine candidate **V3** was formed by formulating **V2** into liposomes. Vaccine candidate **V4** was formed by physically mixing **1–4** with rods.

Four novel delivery systems were tested: poly(methyl acrylate) (PMA) (**V1**), polyleucine (L10) possessing ten amino acid units (**V2**), Leu10 formulated in liposomes (**V3**) and rod-shaped polymeric nanoparticles (rods) built from polystyrene-poly(*N*-isopropylacrylamide)-poly(*N*-dimethylacrylamide) (**V4**) for PCV2 vaccine delivery. In addition, the epitopes adjuvanted with powerful but toxic complete Freund’s adjuvant (CFA) (**V0**) were used as a positive control formulation (Figure 1). Polymers have been of interest for drug/vaccine delivery due to their customizable physicochemical properties, controllable stability in vivo, relative safety and efficacy of cargo delivery to desired cells/tissues [28]. These features allow them to be widely used as immunostimulants for vaccine delivery [29,30], including veterinary vaccines [31,32,33,34]. These polymers have been applied as a nanocarrier or have been directly conjugated to an antigen. The first polymer-peptide antigen conjugation approach was reported in 2010, where J14 epitope (a B cell epitope) was conjugated to dendritic polyacrylate polymer [35]. The conjugate self-assembled into nanoparticles that were efficient in eliciting antibody formation after single-dose subcutaneous and intranasal treatment, in a size dependent manner, and were also shown to be opsonic against all tested GAS clinical isolates [29].

Poly(hydrophobic amino acid) (pHAA), a sequence of repeating units of hydrophobic amino acids that can form fully biodegradable nanoparticles, was tested as a vaccine carrier in mice upon conjugation with a peptide antigen [36]. PADRE-J8 (B cell epitope derived from GAS M protein and universal T cell epitope) was incorporated into a pHAA sequence built based on a variety of hydrophobic amino acids [36,37,38]. The resulting amphiphilic compounds were self-assembled into nanoparticles (10–30 nm), which self-assembled in larger chain-like aggregates. Polyleucine-based nanoparticles induced the maturation of antigen presenting cells (APCs) in vitro and triggered the highest titers of opsonic antibodies among the tested sequences in mice. In addition, the nanoparticles greatly reduced the bacterial burden in mice challenged with the M1 GAS strain without inducing potentially damaging soluble inflammatory mediators. Upon conjugation with hookworm epitope, the polyleucine system was also effective in inducing protective immune responses in intraperitoneally and orally vaccinated hookworm-challenged mice [24,25,39]. Therefore, polyleucine was also selected as a PCV2 vaccine delivery system for this study.

Liposomes were discovered more than half a century ago by Alec Bangham and they have been a mainstay drug delivery system ever since [40]. The liposome lipid bilayer can carry hydrophobic compounds, while the core of the vesicles can encapsulate hydrophilic compounds [41]. Liposome formulations can maximize active ingredients’ therapeutic index, stability and absorption while reducing toxicity and prolonging the biological half-life of the encapsulated compound [41,42]. Antigens encapsulated within liposomes are protected against degradation in vivo and are preferentially taken up by APCs. Cationic liposomes possess immune potentiating properties that alleviate their appeal compared to negatively charged or neutral liposomes, as their positive charge allows the cationic liposomes to interact more efficiently with the immune cells’ anionic membranes [43,44]. Therefore, APCs, such as macrophages and dendritic cells (DCs), preferentially interact with cationic liposomes when compared to neutral or anionic liposomes. Moreover, delivery of polyleucine antigen conjugates with liposomes has been recently tested [45]. Thus, liposomes in combination with polyleucine-based delivery system have been also selected for PCV2 vaccine formulation.

Spleen macrophages are important scavenger cells involved in viral clearance from a host [46]. Recently, rod-shaped polymeric nanoparticles have been reported to have the tendency to accumulate in the spleen systemic administration, especially when produced in form of rods [47]. Indeed, these rods were able to act as a very effective immune adjuvant once physically mixed with an antigen. Interestingly, the physical mixture of rod-shaped polymeric nanoparticles and antigen was found to be more efficient than their chemical conjugates in inducing antibody formation [48].

Herein, four new PCV2 vaccines (**V1**–**V4**) have been designed, synthesized and formulated (Figure 1). Three PCV2b capsid protein epitopes (**1**–**3**) and universal T-helper epitope (**4**) were conjugated with PMA (**5**–**8**) and mixed to produce **V1**, coupled with Leu10 (**9**–**12**) and mixed to produce **V2**, which was further incorporated into liposomes **V3**. Finally, epitopes **1**–**4** were physically mixed with rod-shaped polymeric nanoparticles to produce **V4** or emulsified with CFA to produce control formulation **V0**. All formulations, **V0**–**V4,** were characterized for their size/morphology and evaluated for the ability to produce antigen-specific antibodies in mice.

## 2. Results

**Figure 2:**
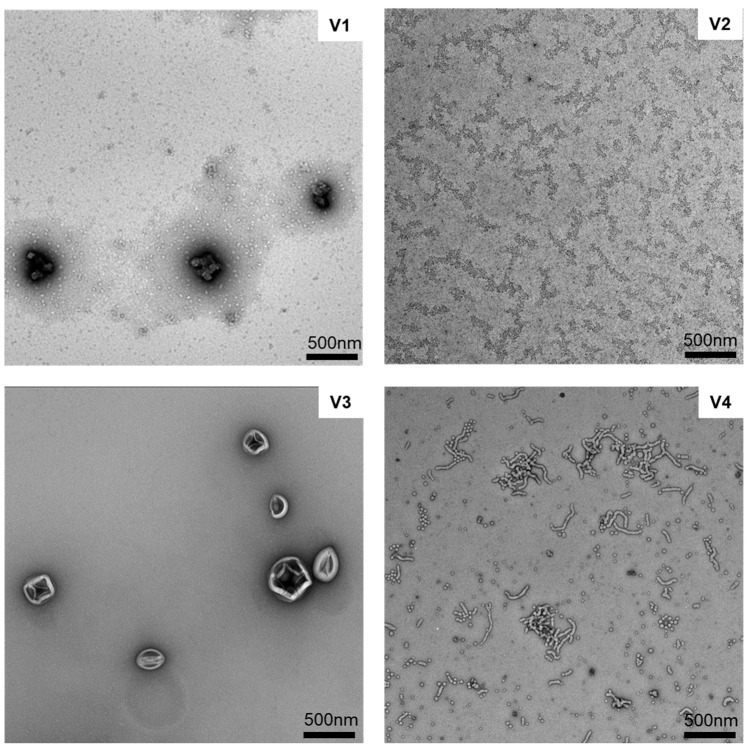
Transmission electron micrographs of PCV2 vaccine candidates **V1**, **V2**, **V3** and **V4**. All compounds were stained with 2% uranyl acetate. Bar = 500 nm.

All PCV2 epitopes and related peptides (**1**–**4**, **P1**–**P4** and **9**–**12**) were synthesized via microwave-assisted Fmoc SPPS [49]. Conjugates **5**–**8** were produced by the conjugation of alkyne-modified antigens (**P1**–**P4**) and PMA using a copper catalyzed Huisgen1,3-dipolar cycloaddition “click” reaction [50]. Following the click reaction, conjugates **5–8** were mixed together and self-assembled via solvent exchange method (DMF-water) and extensively dialyzed for 3 days against water to remove unreacted peptide and residual copper to produce **V1** (Supplementary Figure S3) [50]. Vaccine candidate **V1** (in PBS) formed narrowly polydisperse spherical nanoparticles (DLS analysis: 280 ± 8 nm; PDI = 0.23 ± 0.01) with a positive zeta potential (43.7 ± 0.7 mV) (Table 2, Figure 2, Supplementary Figure S2). Vaccine candidate **V2**, the mixture of peptides **9**–**12**, formed broadly polydisperse, aggregate chain-like nanoparticles (DLS analysis: 86 ± 19 nm, 363 ± 54 nm; PDI = 0.57 ± 0.18) with a positive zeta potential (34.0 ± 0.1 mV) upon simple dissolution in PBS at pH = 7.4 (Table 2, Figure 2, Supplementary Figure S2). The formation of chain-like aggregated nanoparticles for polyleucine conjugates were reported previously [36]. Vaccine candidate **V3** was produced by incorporation of vaccine candidate **V2** into liposomes via thin lipid hydration (precisely, peptides **9**–**12** were incorporated into liposomes during thin layer formation). **V3** formed globular nanoparticles with relatively narrow polydispersity (206 ± 3 nm, PDI = 0.26 ± 0.01) and a positive zeta potential (25.0 ± 0.8 mV) upon simple dissolution in PBS at pH = 7.4 (Table 2, Figure 2, Supplementary Figure S2). Vaccine candidate **V4** was produced by the simple mixing of antigens **1**–**4** with rods in PBS. Vaccine candidate **V4** formed broadly polydisperse nanoparticles as expected for rod-shaped particles (158 ± 15 nm, 503 ± 55 nm, 5200 ± 180 nm; PDI = 0.42 ± 0.07) with a positive zeta potential (11.9 ± 0.7 mV) upon simple dissolution in PBS at pH = 7.4 (Table 2, Figure 2, Supplementary Figure S2).

**Figure 3:**
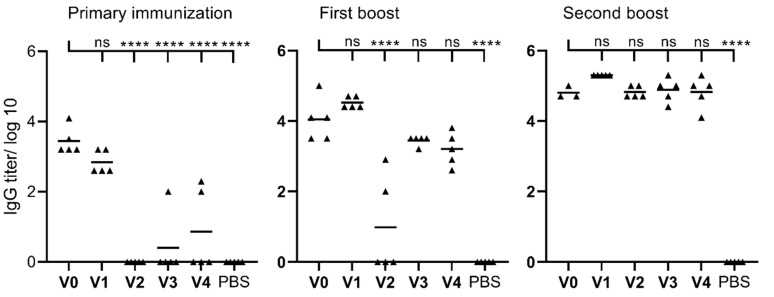
Sera IgG titers of mice immunized with **V0**–**V4** subunit vaccines after the first, second and third immunizations, as analyzed by enzyme-linked immunosorbent assay. Statistical analysis was performed using one-way ANOVA followed by Tukey’s post-hoc test (ns > 1; ****, *p* < 0.0001). PBS: phosphate-buffered saline.

To test vaccine candidate efficacy, mice were immunized subcutaneously with **V0**–**V4** and PBS (Figure 3). **V0** served as a positive control, while PBS was used as a negative control. After the first immunization, significant antibody production against PCV2 antigens was detected in mice immunized with CFA-adjuvanted antigens (**V0**) and PMA-conjugated antigens (**V1**). Notably, the titers induced by the positive control were not significantly higher than those of **V1**. Following second immunization, IgG titters produced by **V1** increased significantly and exceeded these induced by the positive control; however, the difference was not statistically significant. Vaccines **V2** and **V3** also produced high IgG titters, while **V2** was poorly effective after second immunization. Interestingly, after the second boost, all vaccine candidates (**V1**–**V4**) induced IgG production in mice in similar levels to the positive control (CFA-adjuvanted formulation **V0**) (Figure 3).

## 3. Discussion

Systemic humoral immunity, measured as serum antigen-specific antibody titers, has shown strong correlation with protective immunity against PCV2 in pigs [51]. As peptide antigens are not, or are poorly, immunogenic alone (do not induce efficient systemic humoral immunity), a proper vaccine delivery system/adjutant must be incorporated into vaccine formulation. Thus, we investigated four self-adjuvanting delivery systems for inducing antigen-specific antibody responses and compared their efficacy with the gold standard powerful adjuvant, CFA. CFA-based formulation **V0** elicited high IgG titers even after single immunization; however, two mice in this group died at day 65, which made **V0** applicability as a vaccine candidate unacceptable. While single immunization with CFA is usually safe for animals, CFA is also well-known for its reactogenicity and excessive pro-inflammatory responses that can lead to uncontrolled tissue damage [52]. The mice immunized with **V1**–**V4** vaccine candidates did not show any adverse effects, as expected. The delivery systems used in these vaccine formulations have been previously proven to be safe [24,25,48]. Interestingly, the vaccine candidates have very distinct abilities to induce antibody production, especially after the first two vaccinations.

The immune responses generated against nanoparticles (all vaccine candidates **V1**–**V4** were produced in nanoparticles form) are very often correlated with size and shape of particles [53,54,55]. Typically, smaller nanoparticles are more immunogenic [56,57]. The small nanoparticles can easily travel to lymph nodes (without participation of peripheral DCs), and the nodes are the fighting core of the human immune system [56]. In contrast, larger particles have the potential to create a depot effect, keeping the antigen at the injection site and lengthening the time the immune cells are exposed to the vaccine, boosting the immunogenic activity [16]. Thus, nanovaccines have been widely used to target variety of diseases [58,59,60]. **V1**–**V4** all formed nanoparticles, primarily in the range of 200 **to** 500 nm, with some aggregates, as observed by DLS and TEM. Thus, there is not any apparent difference between the vaccines’ particle size. However, while the size of **V1**–**V4** particles was similar, their shapes were significantly different. **V1** formed spherical nanoparticles, **V2** chain-like aggregates of very small nanoparticles, **V3** typical liposomes, i.e., spherical nanoparticles, and **V4** was produced based on rod-shaped nanoparticles, as can be seen on TEM images. Morphology influences antigen presentation by APCs, as well as intracellular particle processing [53]. Both spherical and rod-like particles have been reported to induce superior immunity in comparison to other shapes and when compared to each other [48,61,62]. For example, rod-shaped nanoparticles have a higher tendency to accumulate in the spleen, thereby increasing uptake by the immune system [47] and triggering a strong immune response [48,62,63]. However, only **V1** induced high antibody titters after a single immunization. Thus, the shape of nanoparticles was not associated with their immunogenicity. Finally, surface charge also plays an important role in cellular processing of nanoparticles; uptake by APCs increases with increasing nanoparticle surface charge [64]. As immune cell membranes are anionic in nature, the highly positive surface of **V1** (44 mV) may have facilitated stronger interaction, explaining the stronger and faster stimulation of humoral responses (IgG production) [43]. However, positively charged nanoparticles **V2** (34 mV) induced lower antibody titers upon second immunization than weakly positive **V3** (25 mV) and **V4** (12 mV).

Interestingly, we have previously reported that PMA-based nanoparticulate vaccines were less effective than rod-based when these nanoparticles were applied for GAS antigen delivery (PADRE-J8). Rod-shaped polymeric nanoparticles in physical mixture with PADRE-J8 elicited significantly higher antibody titers than CFA-adjuvanted antigen [48]. However, PMA conjugates have been also reported to be effective after a single immunization in a similar manner as reported here for formulation **V1** [65].

Both **V1** and **V4** were polymeric-based vaccines. Nevertheless, the polymer in **V1** was chemically conjugated to antigen epitopes, whereas the polymeric rods in **V4** were only physically mixed with antigen epitopes. This may suggest that chemical conjugation was preferable over physical mixture in triggering potent immune responses. However, the same rods in physical mixture with antigen have been reported to be more immunogenic than the conjugated equivalent [48]. Finally, the ability of polyleucine to be quantitatively entrapped in liposomes was previously reported [45]; however, this entrapment did not result in enhanced immunity, while here **V3** clearly induced higher antibody titers than **V2** after second immunization.

However, it must be emphasized that. following third immunization. all particles were practically equally effective in inducing antibody titers. Thus, the observed differences between formulations were present only following initial immunizations. It is important to stress that **V1** elicited the quickest antibody response, even at a single dose, similarly to the strong but toxic CFA (**V0**). Most currently available PCV2 vaccines require administration of a single dose, thus **V1** is the most promising vaccine candidate for further optimization and examination, including immunization of pigs and PCV2 challenge.

## 4. Materials and Methods

### 4.1. Chemicals

All chemicals used were analytical grade. Protected 9-fluorenylmethoxycarbonyl (Fmoc) amino acids and rink amide p-methylbenzhydrylamine (MBHA) resin were purchased from Novabiochem (Lau-felfingen, Switzerland). 1-[Bis(dimethylamino)methylene]-1H-1,2,3-triazolo [4,5-b]pyridinium-3-oxide hexafluorophosphate and hexafluorophosphate azabenzotriazole tetramethyl uronium (HATU) was purchased from Mimotopes (Melbourne, Australia). *N,N*-diisopropylethylamine (DIPEA), *N,N*-dimethylformamide (DMF), dicholoromethane (DCM), piperidine, trifluroacetic acid (TFA) and acetonitrile were purchased from Merck (Hohbrunn, Germany). Pentanoic acid, triisopropylsilane (TIPS) and phosphate buffered saline (PBS) were obtained from eBioscience (California, USA). Rod-shaped polymeric nanoparticles (polystyrene-poly(*N*-isopropylacrylamide)-poly(*N*-dimethylacrylamide) were synthesized as reported previously [47]. C57BL/6 mice were purchased from The University of Queensland Biological Resources (UQBR) (Queensland, Australia).

### 4.2. Equipment

Shimadzu reverse-phase HPLC instruments (Shimadzu Corp, Kyoto, Japan) equipped with Vydac C4 and C18 columns were used to analyze and purify the peptides. An ESI-MS electrospray ionization mass spectrometer (Sciex API-3000, Sciex, Vaughan, ON, Canada) was used to confirm structure and purity. A Zetasizer Nano (Malvern Instruments, Worcestershire, UK) was used to measure the particle size distribution of self-assembled vaccines via dynamic laser scattering. A BMG CLARIOStar fluorescence plate reader (BMG LABTECH, Cary, NC, USA) was used to assess ELISA. Mathematical and statistical presentation of the data was conducted using Graphpad Prism v8.3 (GraphPad Software, Inc, San Diego, CA, USA). Electron micrographs of self-assembled vaccine compounds were captured with a JEM-1010 transmission electron microscope (HT7700 Exalens, Hitachi Ltd., Jeol Ltd., Tokyo, Japan).

### 4.3. Synthesis of Peptides, Conjugates and Formulations

Peptides **1**–**4,** pentynoic acid modified **1**–**4 (P1**–**P4)** and **9**–**12** were synthesized using microwave-assisted Fmoc solid-phase peptide synthesis (SPPS) following a previously established protocol [49]. They were purified using preparative reverse-phase high-performance liquid chromatography (RP-HPLC), and their structure and purity (>95%) were confirmed using electrospray ionization mass spectroscopy (ESI-MS) and analytical RP-HPLC (Supplementary Figure S1).

Peptide **1** (SRTIGYTVKKTTVRTPSWNVDM-KK). Yield: 18%. MW: 2582.0; ESI-MS: [M + 2H^+^]^2+^ *m/z* 1291.5 (found 1291.5), [M + 3H^+^]^3+^ *m/z* 861.3 (found 861.3), [M + 4H^+^]^4+^ *m/z* 646.2 (found 646.2). HPLC: C18 column, 0–100% Solvent B (acetonitrile 90%, water 10%, acetic acid 1%), Solvent A (water 99%, acetic acid 1%). Retention time = 15.00 min. Purity 99%.

Peptide **2** (SNPLTVPFEYYRIRKVKVE-KK). Yield: 17%. MW: 2379.8; [M + 2H^+^]^2+^ *m/z* 1190.9 (found 1191.0), [M + 3H^+^]^3+^ *m/z* 794.0 (found 794.3), [M + 4H^+^]^4+^ *m/z* 596.4 (found 596.0). HPLC: C18 column, 0–100% Solvent B, Solvent A. Retention time = 22.58 min. Purity 98%.

Peptide **3** (KPVLDRTIDYFQPNNKR-KK). Yield: 16%. MW: 2145.5; [M + 2H^+^] ^2+^ *m/z* 1073.7 (found 1069.3), [M + 3H^+^] ^3+^ *m/z* 716.1 (found 713.2), [M + 4H^+^] ^4+^ *m/z* 537.3 (found 535.2). HPLC: C4 column, 0–100% Solvent B (acetonitrile 90%, water 10%, acetic acid 1%), Solvent A (water 99%, acetic acid 1%). Retention time = 37.20 min. Purity 98%.

Peptide **4** (KLIPNASLIENCTKAEL-KK). Yield: 17%. MW: 2755.3; [M + 2H^+^]^2+^ *m/z* 1378.7 (found 1378.9), [M + 3H^+^]^3+^ *m/z* 919.4 (found 919.6), [M + 4H^+^]^4+^ *m/z* 689.8 (found 689.9). C4 column, 0–100%, Solvent B (acetonitrile 90%, water 10%, acetic acid 1%), Solvent A (water 99%, acetic acid 1%). Retention time = 21.29 min. Purity 99%.

Alkyne-modified peptide **1**, **P1** (CHCCH_2_CH_2_C(O)-SRTIGYTVKKTTVRTPSWNVDM-KK). Yield: 18%. MW: 2620.2; [M + 2H^+^]^2+^ *m/z* 1311.1 (found 1312.0), [M + 3H^+^]^3+^ *m/z* 874.4 (found 875.0), [M + 4H^+^]^4+^ *m/z* 656.1 (found 656.5). HPLC: C18 column, 0–100% Solvent B (acetonitrile 90%, water 10%, acetic acid 1%), Solvent A (water 99%, acetic acid 1%). Retention time = 20.04 min. Purity 99%.

Alkyne-modified peptide **2**, **P2** (CHCCH_2_CH_2_C(O)-SRTIGYTVKKTTVRTPSWNVDM-KK). Yield: 17%. MW: 2417.8; [M + 2H^+^]^2+^ *m/z* 1209.9 (found 1209.7), [M + 3H^+^]^3+^ *m/z* 806.9 (found 806.8), [M + 4H^+^]^4+^ *m/z* 605.5 (found 605.4). C18 column, 0–100% Solvent B (acetonitrile 90%, water 10%, acetic acid 1%), Solvent A (water 99%, acetic acid 1%). Retention time = 23.03 min. Purity 98%.

Alkyne-modified peptide **3**, **P3** (CHCCH_2_CH_2_C(O)-KPVLDRTIDYFQPNNKR -KK). Yield: 16%. MW: 2183.5; [M + 2H^+^] ^2+^ *m/z* 1092.8 (found 1093.1), [M + 3H^+^] ^3+^ *m/z* 729.1 (found 729.1), [M + 4H^+^] ^4+^ *m/z* 546.9 (found 547.0). C4 column, 0–100% Solvent B (acetonitrile 90%, water 10%, acetic acid 1%), Solvent A (water 99%, acetic acid 1%). Retention time = 22.78 min. Purity 98%.

Alkyne-modified peptide **4**, **P4** (CHCCH_2_CH_2_C(O)-KLIPNASLIENCTKAEL-KK). Yield: 17%. MW: 2192.7; [M + 2H^+^]^2+^ *m/z* 1097.3 (found 1097.4), [M + 3H^+^]^3+^ *m/z* 731.9 (found 731.9), [M + 4H^+^]^4+^ *m/z* 549.2 (found 549.2). C18 column, 0–100% Solvent B (acetonitrile 90%, water 10%, acetic acid 1%), Solvent A (water 99%, acetic acid 1%). Retention time = 22.82 min. Purity 99%.

Peptide **9** (LLLLLLLLLL-SRTIGYTVKKTTVRTPSWNVDM-KK). Yield: 18%. MW: 3713.6; [M + 2H^+^]^2+^ *m/z* 1857.8 (found 1858.2), [M + 3H^+^]^3+^ *m/z* 1238.9 (found 1239.1), [M + 4H^+^]^4+^ *m/z* 929.4 (found 929.6). C4 column, 0–100% Solvent B (acetonitrile 90%, water 10%, acetic acid 1%), Solvent A (water 99%, acetic acid 1%). Retention time = 32.57 min. Purity 99%.

Peptide **10** (LLLLLLLLLL- SNPLTVPFEYYRIRKVKVE-KK). Yield: 17%. MW: 3512.4; [M + 2H^+^]^2+^ *m/z* 1757.2 (found 1756.8), [M + 3H^+^]^3+^ *m/z* 1171.8 (found 1171.5), [M + 4H^+^]^4+^ *m/z* 879.1 (found 878.9). C4 column, 0–100% Solvent B (acetonitrile 90%, water 10%, acetic acid 1%), Solvent A (water 99%, acetic acid 1%). Retention time = 36.28 min. Purity 98%.

Peptide **11** (LLLLLLLLLL-KPVLDRTIDYFQPNNKR-KK). Yield: 16%. MW: 3278.0; [M + 2H^+^] ^2+^ *m/z* 1640.0 (found 1639.8), [M + 3H^+^] ^3+^ *m/z* 1094.0 (found 1093.5); [M + 4H^+^] ^4+^ *m/z* 820.5 (found 820.4). C4 Column, 0–100% Solvent B (acetonitrile 90%, water 10%, acetic acid 1%), Solvent A (water 99%, acetic acid 1%). Retention time = 36.25 min. Purity 98%.

Structure **12** (LLLLLLLLLL-KLIPNASLIENCTKAEL-KK). Yield: 17%. MW: 3287.2; [M + 2H^+^]^2+^ *m/z* 1644.6 (found 1644.8), [M + 3H^+^]^3+^ *m/z* 1096.7 (found 1096.4, [M + 4H^+^]^4+^ *m/z* 822.8 (found 822.9). C4 column, 0–100% Solvent B (acetonitrile 90%, water 10%, acetic acid 1%), Solvent A (water 99%, acetic acid 1%). Retention time = 32.75 min. Purity 99%.

### 4.4. Synthesis of Polymer Conjugates ***5***–***8***

**P1**–**P4** were conjugated to azide-modified PMA using a copper(I)-catalyzed alkyne-azide cycloaddition (CuAAC) click reaction [50]. Copper wire was submerged in concentrated sulfuric acid for 1 min, then washed on a glass filter funnel with MilliQ water (5 times) and methanol (5 times) before being dried under a stream of nitrogen. 10 mg PMA and 20 mg 4-pentynoyl peptide moiety, copper wire (10 mg) and DMF (2 mL) were added to the flask and stirred. The click reaction was terminated after 12 h when the solution color turned to greenish-blue. The resulting solution was filtered through a Cameo^®^ syringe filter with PTFE membrane (pore size: 0.45 μm, volume: 12 mL). The polymer-peptide conjugate was self-assembled by solvent exchange (DMF-water) using a syringe pump. A DMF solution containing the polymer peptide conjugate (2 mL) was slowly added to the water (4 mL) over 3 h. The conjugate was dialyzed for 3 days to remove unreacted peptide, copper and organic solvent. The resulting conjugate nanoparticles (**5–8**) were analyzed upon freeze drying by element microanalysis to determine the antigen substitution ratio as previously reported [50], based on comparison of the theoretical N/C (nitrogen carbon ratio) and the N/C obtained from elemental analysis (Supplementary Figure S3) and characterized by DLS and TEM (Figure 2 and Supplementary Figure S2).

### 4.5. Preparation of Vaccine Candidate ***V0***

Vaccine candidate **V0** was freshly prepared before each immunization by emulsification of **1**–**4** with CFA. Thus, peptides **1**–**4** (0.25 mg of each) dissolved in PBS (0.5 mL) was emulsified with 0.5 mL of CFA solution.

### 4.6. Preparation of Vaccine Candidate ***V1***

The solution of conjugates **5**–**8** (0.5 mg of each) in DMF (0.5 mL each) were mixed together and slowly added to the water (4 mL) using a syringe pump over 2 h. The mixture of conjugates was dialyzed for 3 days and then concentrated (1.1 mg/mL of **5**–**8** in water). Finally, 10X PBS (0.1 mL) was added to the aqueous solution of **5**–**8** (0.9 mL) to produce **V1** (1.0 mg/mL of **5**–**8** in PBS).

### 4.7. Preparation of Vaccine Candidate ***V2***

Peptides **9**–**12** (0.25 mg each) were dissolved in PBS (1.0 mL). The solution of peptides (1 mg/mL) was vortexed for 1 min.

### 4.8. Preparation of Vaccine Candidate ***V3***

Liposomes were prepared by thin lipid hydration. Chloroform (1 mL) was used to dissolve DPPC (4 mg), DDAB (0.017 mg) and cholesterol (1.05 mg) (at a molar ratio of 2:0.01:1). The peptides **9**–**12** (0.25 mg of each peptide) were dissolved in 1 mL of methanol to form a clear solution and added to the lipid solution. The solvents were then slowly evaporated under reduced pressure using a rotatory evaporator to produce a film on the inner surface of a round bottom flask. Residual solvents were removed under vacuum overnight. The lipids were rehydrated with 1 mL of water and swirled gently to produce liposomes. The emulsion was extruded 21 times in both directions using a 200 nm pc membrane filter to create homogenous liposomes. Formulation in PBS was achieved by adding 0.1 mL of 10X PBS into 0.9 mL of the liposomal formulation. The final concentration of **9**–**12** in liposomes was 1 mg/mL.

### 4.9. Preparation of Vaccine Candidate ***V4***

Peptides **1**–**4** (0.25 mg each) were dissolved in PBS (0.5 mL) and gently mixed with the solution of rods (1 mg in 0.5 mL of PBS).

### 4.10. Dynamic Light Scattering

Individual vaccine candidates **V1**–**V4** (1 mg/mL in PBS) were analyzed using DLS to measure particle size (zeta intensity) and PDI. Measurements were taken at 25 °C and 173° light scattering using a Malvern Zetasizer Nano ZP instrument with Malvern Zetasizer Analyser 6.2 software (Malvern, Worcestershire, UK).

### 4.11. Transmission Electron Microscopy

Particle imaging was captured using a JEM-1010 TEM (HT7700 Exalens, HITACHI Ltd., JEOL Ltd., Japan) operated at 80 kV, using negative staining. Samples of **V1**–**V4** (1:2 dilution of samples prepared for DLS analysis) were applied to glow-discharged carbon-coated copper 200 mesh grids (Ted Pella) and stained with 2% uranyl acetate.

### 4.12. Immunization

C57/BL6 female mice (six weeks old) were used for immunization experiments [35,66]. Mice, housed in cages under sterile conditions, were evenly divided into four groups (**V1**–**V4**), and positive (**V0**) and negative control (PBS) groups, with five mice per group. Each mouse received 50 μg of the vaccine candidate (in the respective formulations) in 50 μL of PBS, except for mice in the negative control group, that just received 50 μL PBS. Immunizations were carried out on day 1, 22 and 43. Blood samples were collected via tail bleed on days 0, 21 and 42. Blood was centrifuged for 10 min at 8000 rpm. The supernatant serum was then transferred into sterile tubes and stored at −80 °C for further use. All animal experiments were approved by The University of Queensland Animal Ethics Committee (AEC), AEC Approval number: 2017/AE000069.

### 4.13. Antibody Titer Detection

IgG levels were examined by enzyme-linked immunosorbent assay [45]. In preparation, 96-well microtiter plates were coated with carbonate coating buffer comprising 50 μg of mixture **1**–**3** as antigens. To minimize nonspecific binding, the plates were subsequently blocked with 5% skim milk. Serum samples were serially diluted in 0.5% skim milk, starting at 1:100, down the plate. Afterwards, secondary antibody (33 μL of horseradish peroxide-conjugated anti-mouse IgG) in 100 mL of 0.5% skim milk was added to the plates. The plates were then incubated with 100 μL of OPD substrate (o-phenylenediamine dihydrochloride substrate tablet) for 20 min at room temperature. Absorbance was measured at 450 nm using a Spectra Max microplate reader (Molecular Devices, San Jose, USA).

### 4.14. Statistical Analysis

Statistical analysis of antibody titers between groups was performed using one-way analysis of variance (ANOVA) followed by Tukey’s multiple comparisons test. GraphPad Prism 7.03 software (GraphPad Software Inc., La Jolla, USA) was used for statistical analysis. Differences were considered significant at *p* < 0.05.

## 5. Conclusions

All currently approved PCV2 vaccines use attenuated whole PCV2a pathogen or its fragments, and thus do not cover other subtypes of PCV. We developed novel peptide-based subunit PCV2b vaccine candidates that elicited high IgG antibody titers in mice. All our vaccine delivery systems were as effective as the powerful but toxic commercial adjuvant (CFA) after 3 immunizations. However, only the poly(methyl acrylate)-based delivery system induced superior antibody titers after a single immunization, showing its advantage over other systems. To ultimately prove efficacy of the vaccines, immunization and challenge studies in pigs are still required. Nevertheless, our strategy opens the door for the application of multiepitope peptide-based subunit vaccines against PCV2. As many farms worldwide are burdened by PCV2, the development of more effective vaccines against PCV would be a great economic gain to the swine industry.

## Figures and Tables

**Table 1:** Commercially available PCV2 vaccines.

Vaccine	Antigen	Adjuvant	Administration
Circovac^®^	Inactivated PCV2a	Light paraffin oil	Single dose, intramuscular (IM) for piglets older than 3 weeks or healthy female pigs of breeding age; or two doses, prior to breeding for gilts and sows.
Ingelvac CircoFlex^®^	Capsid PCV2a	Cross-linked carbomer-polymer	Single dose, IM
Circumvent^®^	Capsid PCV2a	D1-α-tocopherol + liquid paraffin	Two doses, IM (3 weeks old)
Porcillis^®^ PCV	Capsid PCV2a	D1-α-tocopherol + liquid paraffin	Two doses, IM (3 days old) or Single dose, IM (3 weeks old)
Fostera™ PCV (Suvaxyn^®^ PCV2 one dose™)	Inactivated chimaeric PCV1/2a	Sulpholipo-cyclodextrin in squalane	Single dose, IM (3 weeks old)

**Table 2:** Particle size, PDI and zeta potential of the vaccine candidates.

Vaccine Candidate	Antigen	Adjuvanting Moiety	Size (nm)	PDI	Zeta Potential (mV)
**V1**	Cap41, Cap67, Cap121, P25	PMA	280 ± 8	0.23 ± 0.01	43.7 ± 0.7
**V2**	Cap41, Cap67, Cap121, P25	Leu10	86 ± 19	0.57 ± 0.20	34.0 ± 0.1
			363 ± 54		
**V3**	Cap41, Cap67, Cap121, P25	Leu10, liposome	206 ± 3	0.26 ± 0.01	25.0 ± 0.8
**V4**	Cap41, Cap67, Cap121, P25	rods	158 ± 15	0.42 ± 0.07	11.9 ± 0.7
			503 ± 55		
			5200 ± 180		

## Data Availability

The data presented in this study are available in article and Supplementary Materials.

## Supplementary Materials

The following supporting information can be downloaded at: https://www.mdpi.com/article/10.3390/molecules28052248/s1, Figure S1: HPLC and ESI-MS spectra of **1**–**4**, **P1**–**P4** and **9**–**12**; Figure S2: DLS spectra of vaccine candidates **V1**–**V4** (size distribution by intensity and zeta potential); Figure S3: The curve of the theoretical substitution ratio (1 = 100%, horizontal axis) of peptide **P1**–**P4** to PMA in conjugates **5**–**8** versus N/C ratio (nitrogen carbon ratio of elemental analysis) of conjugates (vertical axis). Experimentally determined N/C by elemental analysis: 0.124 (**5**), 0.170 (**6**), 0.091 (**7**), and 0.204 (**8**).

## Sample Availability

Samples of the compounds **1–12** are available from the authors.
