# Supplementary material for: Multiepitope Subunit Peptide-Based Nanovaccine against Porcine Circovirus Type 2 (PCV2) Elicited High Antibody Titers in Vaccinated Mice
Source: Molecules. 2023 Feb 28;28(5):2248. doi: 10.3390/molecules28052248 (PMC10005372; doi:10.3390/molecules28052248)
Supplement: Supplementary file 1 [file molecules-28-02248-s001.zip › molecules-2217188-supplementary.pdf]

molecules

Supplementary Materials

Multiepitope Subunit Peptide-Based Nanovaccine against Por-Cine Circovirus Type 2 (PCV2) Elicited High Antibody Titers in Vaccinated Mice

Viet Tram Duong [1], Prashamsa Koirala [1], Sung-Po R. Chen [2], Michael J. Monteiro [2], Mariusz Skwarczynski [1,*] and Istvan Toth [1,3,*]

[1] School of Chemistry and Molecular Biosciences, The University of Queensland, St Lucia, QLD 4072, Australia
[2] Australian Institute of Bioengineering and Nanotechnology, The University of Queensland, St Lucia, QLD 4072, Australia
[3] School of Pharmacy, The University of Queensland, Woolloongabba, QLD 4102, Australia
* Correspondence: m.skwarczynski@uq.edu.au (M.S.); i.toth@uq.edu.au (I.T.)

Peptide **1**

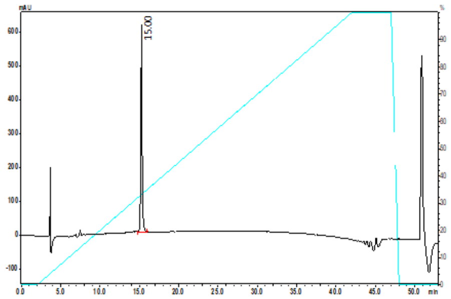

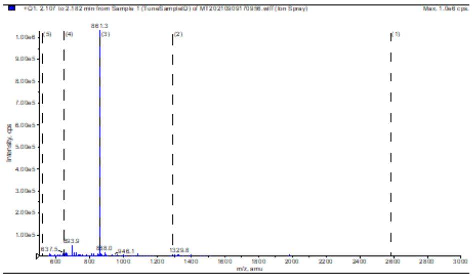

Peptide **2**

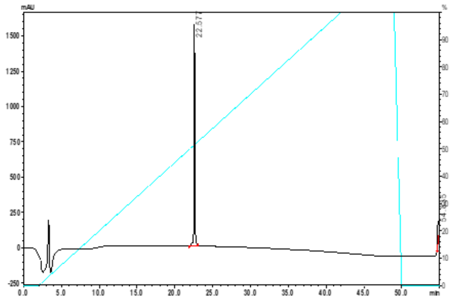

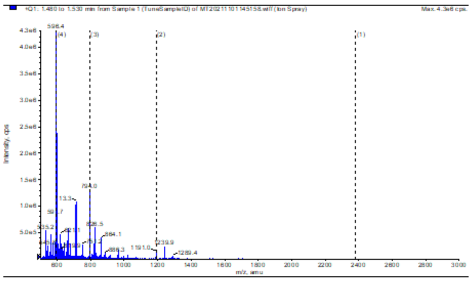

Peptide **3**

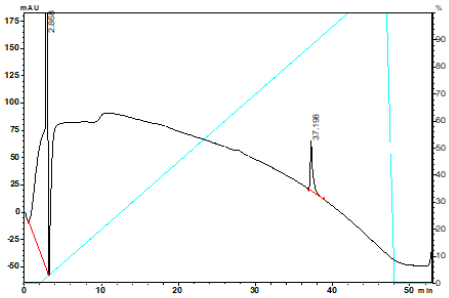

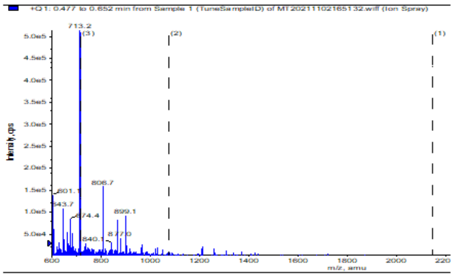

Peptide **4**

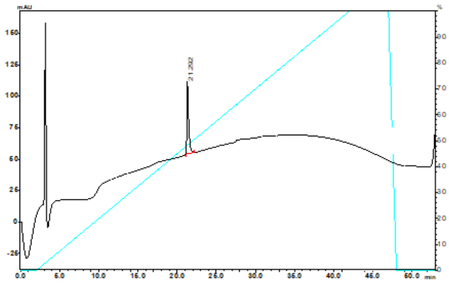

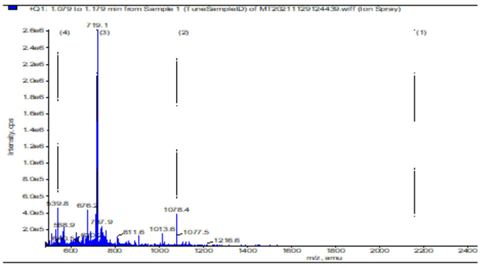

Peptide **P1**

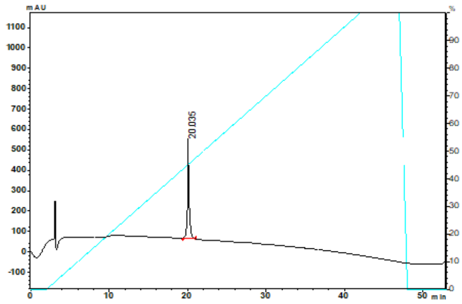

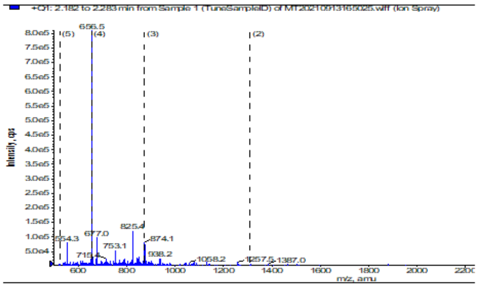

Peptide **P2**

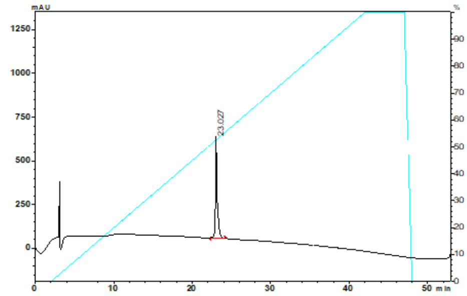

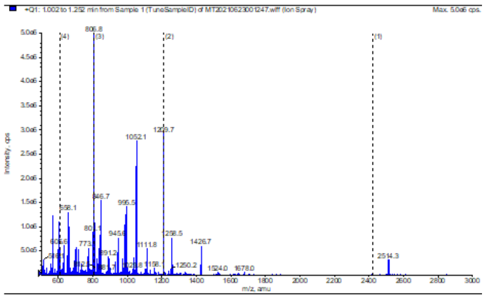

Peptide **P3**

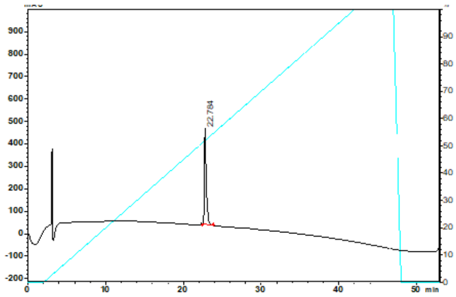

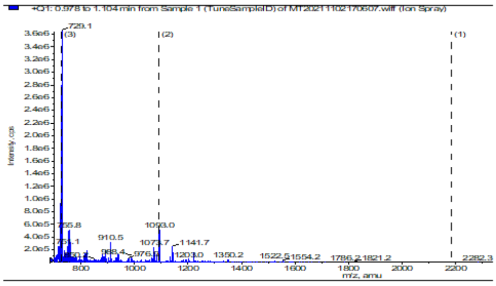

Peptide **P4**

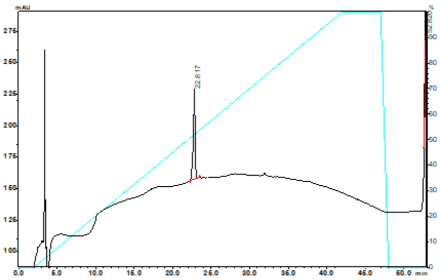

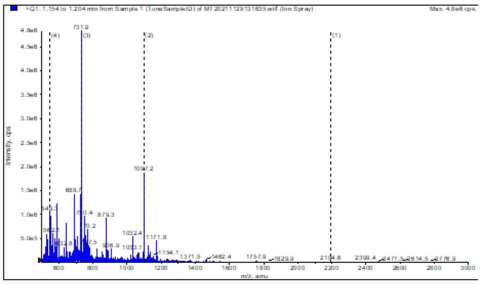

Conjugate **9**

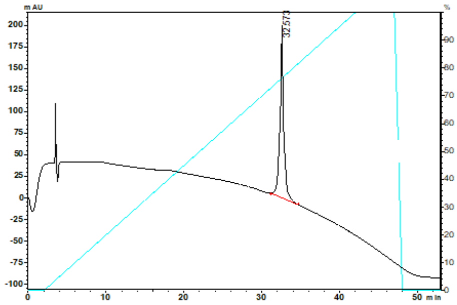

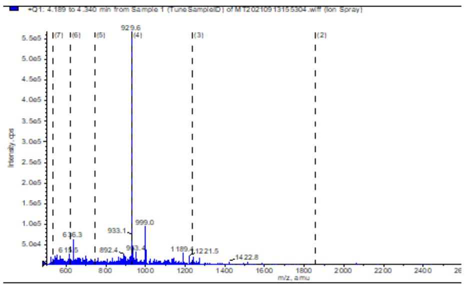

Conjugate **10**

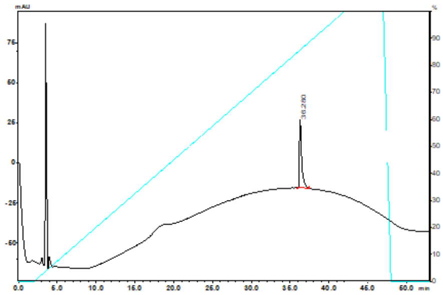

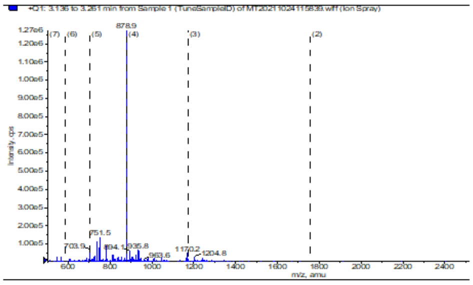

Conjugate **11**

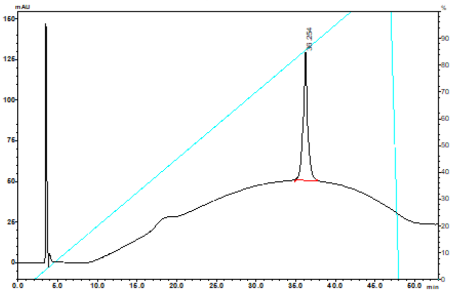

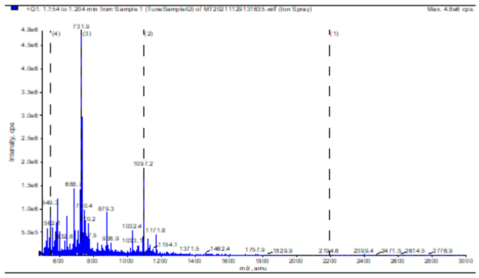

Conjugate **12**

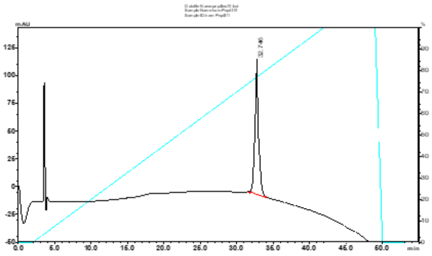

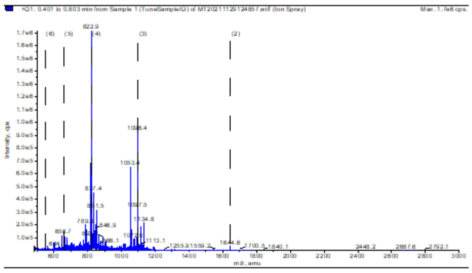

Figure S1. HPLC and ESI-MS spectra of **1–4**, **P1-P4** and **9–12**.

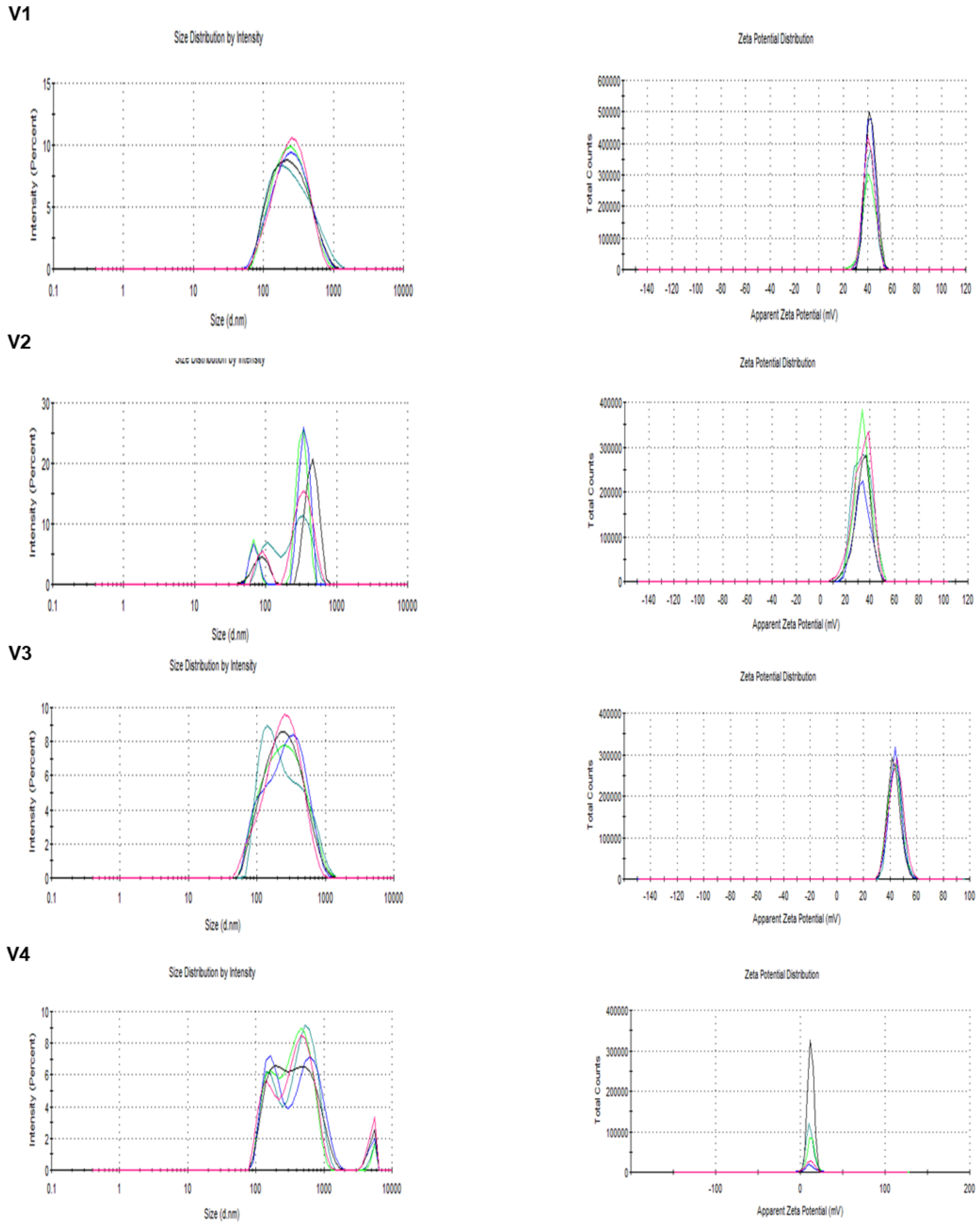

Figure S2. DLS spectra of vaccine candidates **V1-V4** (size distribution by intensity and zeta potential).

Conjugate **5**

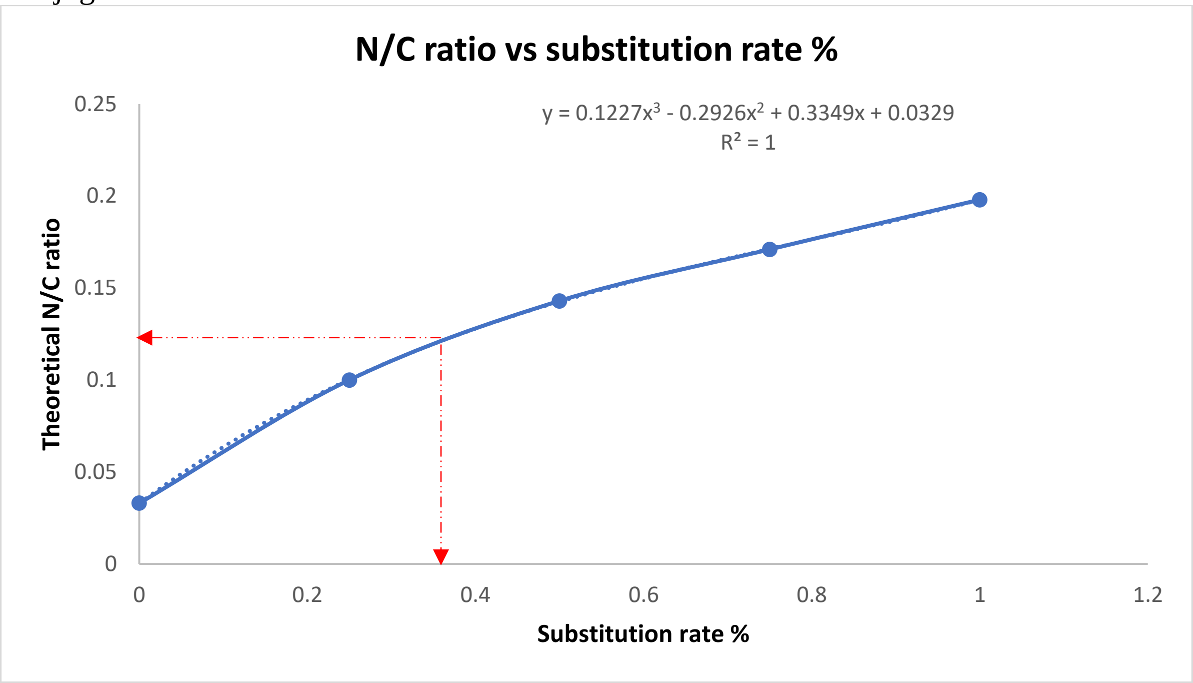

Conjugate **6**

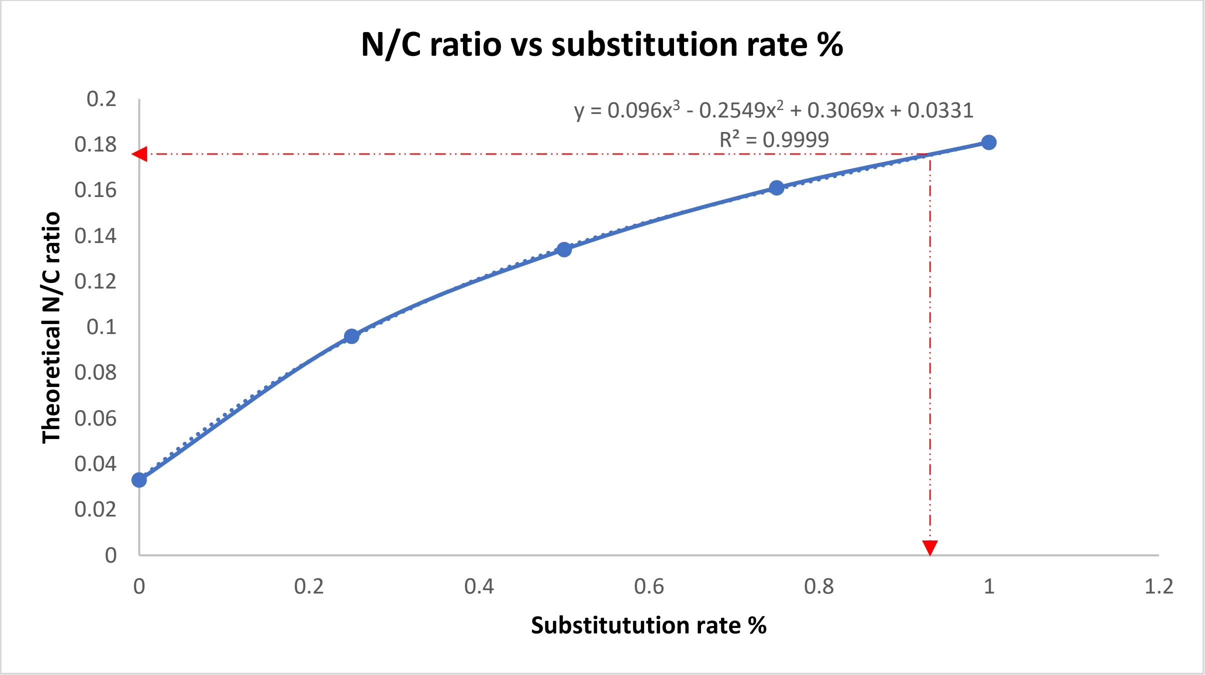

Conjugate **7**

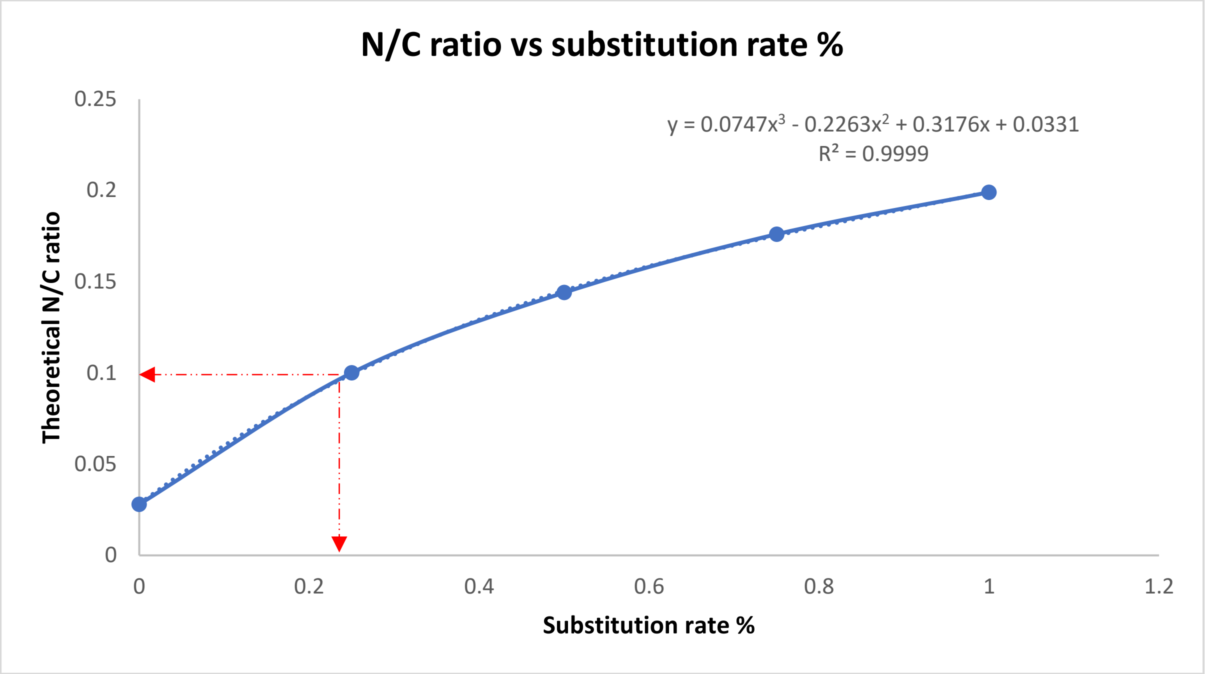

Conjugate **8**

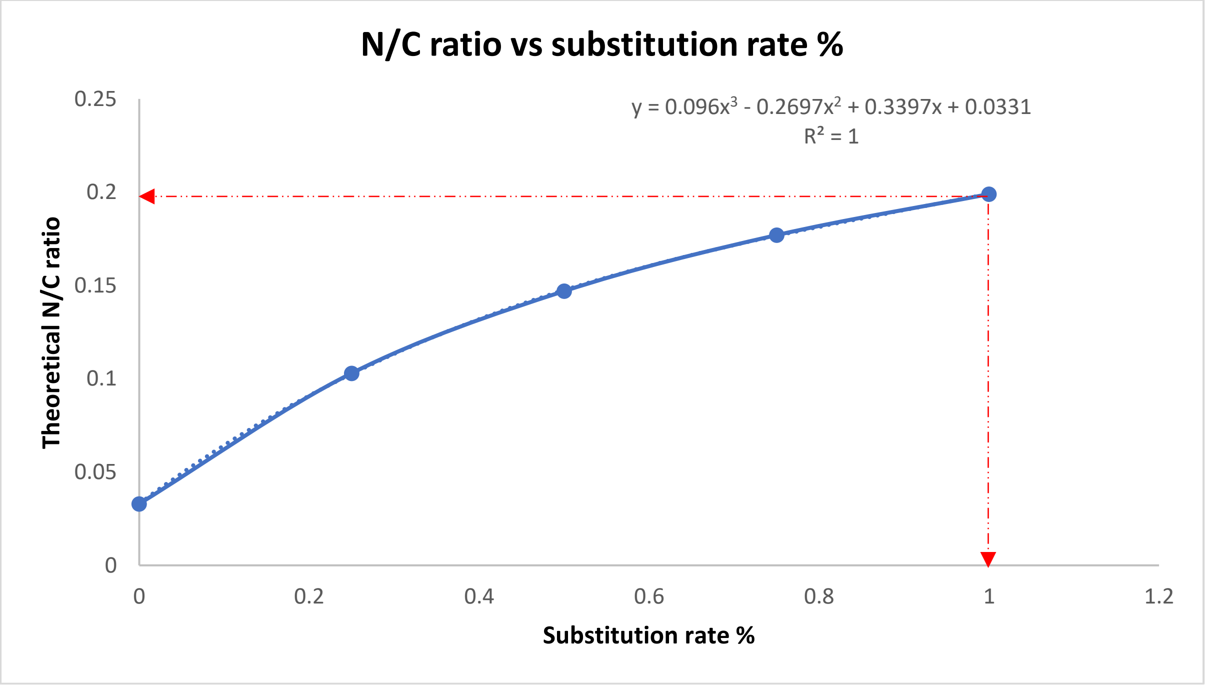

Figure S3. The curve of the theoretical substitution ratio (1 = 100%, horizontal axis) of peptide **P1-P4** to PMA in conjugates **5-8** versus N/C ratio (nitrogen carbon ratio of elemental analysis) of conjugates (vertical axis). Experimentally determined N/C by elemental analysis: 0.124 (**5**), 0.170 (**6**), 0.091 (**7**), and 0.204 (**8**).
